# Expanding horizons of iminosugars as broad-spectrum anti-virals: mechanism, efficacy and novel developments

**DOI:** 10.1007/s13659-024-00477-5

**Published:** 2024-09-26

**Authors:** Qiantong Liu, Yanyun Liu, Tingting Liu, Jinbao Fan, Zanxian Xia, Yingjun Zhou, Xu Deng

**Affiliations:** 1 Xiangya School of Pharmaceutical Sciences, Central South University, Changsha, 410013 Hunan China; 2 School of Life Science, Central South University, Changsha, 410013 Hunan China; 3 Hunan Key Laboratory of Diagnostic and Therapeutic Drug Research for Chronic Diseases, Central South University, Changsha, 410013 Hunan China

**Keywords:** Iminosugars, Broad-spectrum anti-virals, Mode of actions, Structure–activity relationships (SARs)

## Abstract

Iminosugars, a class of polyhydroxylated cyclic alkaloids with intriguing properties, hold promising therapeutic potentials against a broad spectrum of enveloped viruses, including DENV, HCV, HIV, and influenza viruses. Mechanistically, iminosugars act as the competitive inhibitors of host endoplasmic reticular α-glucosidases I and II to  disrupt the proper folding of viral nascent glycoproteins, which thereby exerts antiviral effects. Remarkably, the glycoproteins of many enveloped viruses are significantly more dependent on the calnexin pathway of the protein folding than most host glycoproteins. Therefore, extensive interests and efforts have been devoted to exploit iminosugars as broad-spectrum antiviral agents. This review provides the summary and insights into the recent advancements in the development of novel iminosugars as effective and selective antiviral agents against a variety of enveloped viruses, as well as the understandings of their antiviral mechanisms.

**Graphical Abstract:**

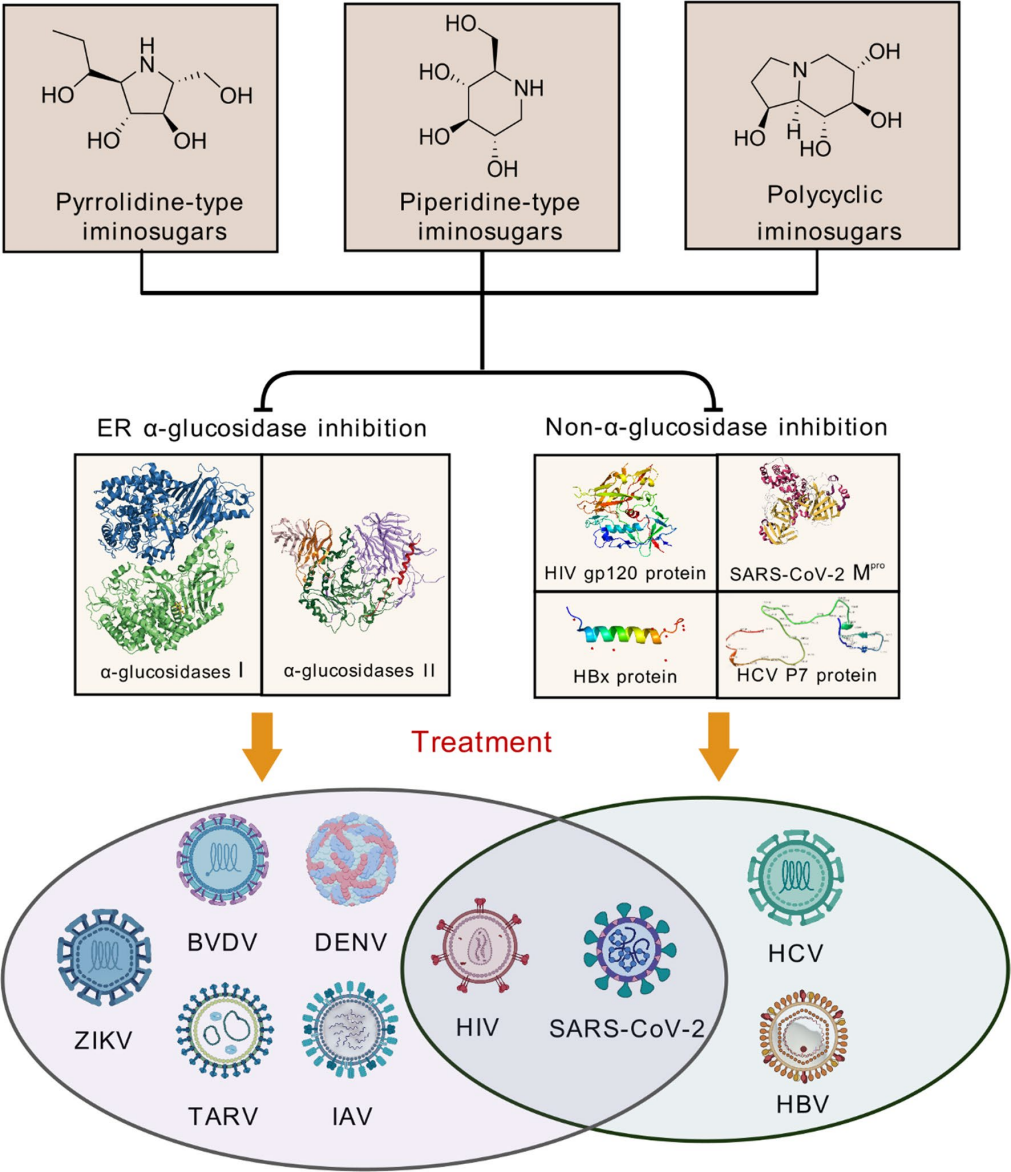

## Introduction

Enveloped viruses, including influenza virus, coronavirus, herpes simplex virus, flavivirus, human immunodeficiency virus (HIV) and filovirus, are featured by a glycoprotein-containing lipid bilayer (envelope) that encapsulates the virion [1]. These viruses are able to evade host immune detection and facilitate entry into the host cell by fusing with the membrane, which have caused several major disease outbreaks and pandemics, including *COVID-19*, thereby severely threatening global public health security and leading to enormous economic losses [2]. Despite numerous drugs have been developed for the treatment of enveloped virus-caused diseases, their long-term efficacy is often undermined by the high mutation rate and rapid emergence of drug resistance [3]. Besides, the constant emergence of new viruses and the increasing frequency of pandemic outbreaks underscores the urgent need for the broad-spectrum anti-virals [4,5]. At present, most approved anti-virals target viral proteins to inhibit specific steps in the viral infection cycle. However, this virus-targeting strategy have some inherent disadvantages, including narrow-spectrum of antiviral activity, vulnerability to develop drug resistance due to the selection pressure, inability to deal with emerging new viruses [6]. In contrast, host-directed therapy (HDT), which targets the host factors that are essential for viral replications or survival, offers a promising approach for the development of broad-spectrum antiviral agents, since viruses use many of the same host proteins (i.e., dihydroorotate dehydrogenase (DHODH), chemokine receptor type 5, inosine monophosphate dehydrogenase, cyclophilins) to replicate [7,8]. Compared to viruses, the host exhibits relatively low genetic variability, rendering host-directed antiviral drugs less likely to induce development of drug resistance. Besides, HDT can mitigate damage to the human body during viral infections by boosting immunity, reducing virus-induced inflammation, and balancing the host antiviral immune responses. It aims to curb the development of drug-resistant mutants, thus slowing down the infections [9]. Among these, the endoplasmic reticulum (ER) α-glucosidase in the nascent glycoprotein folding quality control machinery is crucial for the replication of numerous enveloped viruses [10], which has been regarded as promising HDT for broad-spectrum antiviral therapy.

Iminosugars, a class of polyhydroxylated cyclic alkaloids, mimic carbohydrates in host cells by replacing the endocylic oxygen with nitrogen [11], which have attracted wide interests from both chemistry and biology communities. Typically, iminosugars act as small intestinal α-glucosidase inhibitors and glucose ceramide synthetase inhibitors, thereby interfering the carbohydrate absorption in the small intestine and inhibiting the sphingolipid biosynthesis [12]. Two iminosugar-based drugs, Glyset™ and Zavesca™, have been developed for the treatment of diabetes and type 1 Gaucher diseases in clinic, respectively. Recently, iminosugars have also been shown as competitive inhibitors of *N*-glycan processing α-glucosidases in ER, which were effective against various enveloped viruses [5], including flavivirus [6,13,14] coronavirus [15,16], influenza virus [17].

This review aims to highlight recent advances in the discovery and development of iminosugars as antiviral agents, their structure–activity relationships (SARs), as well as the current understandings of their underlying modes of actions.

## Mode of actions

Given the structural similarity to the endogenous carbohydrates, iminosugars competitively inhibit glucosidases that are critical in glycogenolysis, glycoprotein processing, and saccharide hydrolysis. This inhibition impedes viral glycoprotein processing and glycolipid metabolism, thereby exerting their anti-viral activity and reduce the viral infectivity.

### Iminosugars interfere *N*-linked glycan processing of nascent viral glycoproteins by inhibiting ER α-glucosidases

*N*-Glycosylation, the attachment of a glycan to the amide side chain of the asparagine (Asn) residues in a protein, is the most common form of protein post-translational modifications. This process is essential for the proper folding, trafficking and/or receptor binding of both host and viral glycoproteins, which thereby significantly influences the viral pathogenesis and the immune evasion [18].

Specifically, the common precursor for *N*-glycosylation is the triantennary tetradecasaccharide Glc3Man9GlcNAc2, which possesses three non-reducing carbohydrate terminal branches. In nascent *N*-linked glycoproteins, the distal termini of their *N*-glycans contains three terminal glucose residues, namely Glc-α1,2-Glc-α1,3-Glc, which are removed sequentially. ER α-glucosidase I removes the outermost α1,2-linked glucose, yielding Glc2Man9GlcNAc2. Then, ER α-glucosidase II hydrolyzes the second α-1,3-linked glucose residues to form Glc1Man9GlcNAc2. Subsequently, ER α-glucosidase II hydrolyzes the Glc-α1,3-Man glycosidic linkages to release the third α1,3-linked glucose and generate Man9GlcNAc2. Monoglycosylated glycoproteins (Glc1Man9GlcNAc2) are recognized and retained by the ER lectins, including calnexin and calreticulin, ensuring the correct glycoprotein folding. In case of protein misfolding, further UDP-glycose glycoprotein glucosyltransferase (UGGT)-mediated deglycosylation/re-glycosylation cycles may be initiated [19]. Inhibiting host ER glucosidases I and II interfere with the correct glycoprotein folding in ER, thereby resulting in ER-associated degradation (ERAD) and reducing virion secretion [20].

Since viruses lack their own glycosylation machinery, they rely on host cells to assemble their envelope glycoproteins [3]. All enveloped viruses, with a membrane surrounding the viral capsid, contain glycoproteins, which are thus potentially susceptible to glucosidase inhibitors [21]. T. Block has predicted that viruses that strongly depend on the calnexin/calreticulin pathway for morphogenesis would be sensitive to ER α-glucosidase inhibitors [6,10]. For instance, hepatitis B virus (HBV) and bovine viral diarrhoea virus (BVDV), both of which depending on calnexin for the maturation of viral glycoproteins, are significantly inhibited by α-glucosidase inhibitors [22,25]. Other of medical significance include hepatitis C virus and many haemorrhagic fever-causing flaviviruses (i.e., Dengue virus, West Nile virus and Japanese Encephalitis virus). Recent work has unveiled that individuals with mutations in the mannosyl-oligosaccharide glucosidase (MOGS) genes, crucial for *N*-glycan trimming, showed reduced susceptibility to HIV and influenza viruses [23]. However, these mutations do not affect the susceptibility of non-enveloped viruses (i.e., adenovirus, PV1, and vaccinia virus) that do not rely on glycosylation for entry or egress [24]. These findings underscore the pivotal role of glycosylation in the pathogenesis of various enveloped viruses, including Hepatitis B viruses (HBV) [25], influenza viruses [26], and HIV. This represents a promising antiviral strategy.

**Fig. 1:**
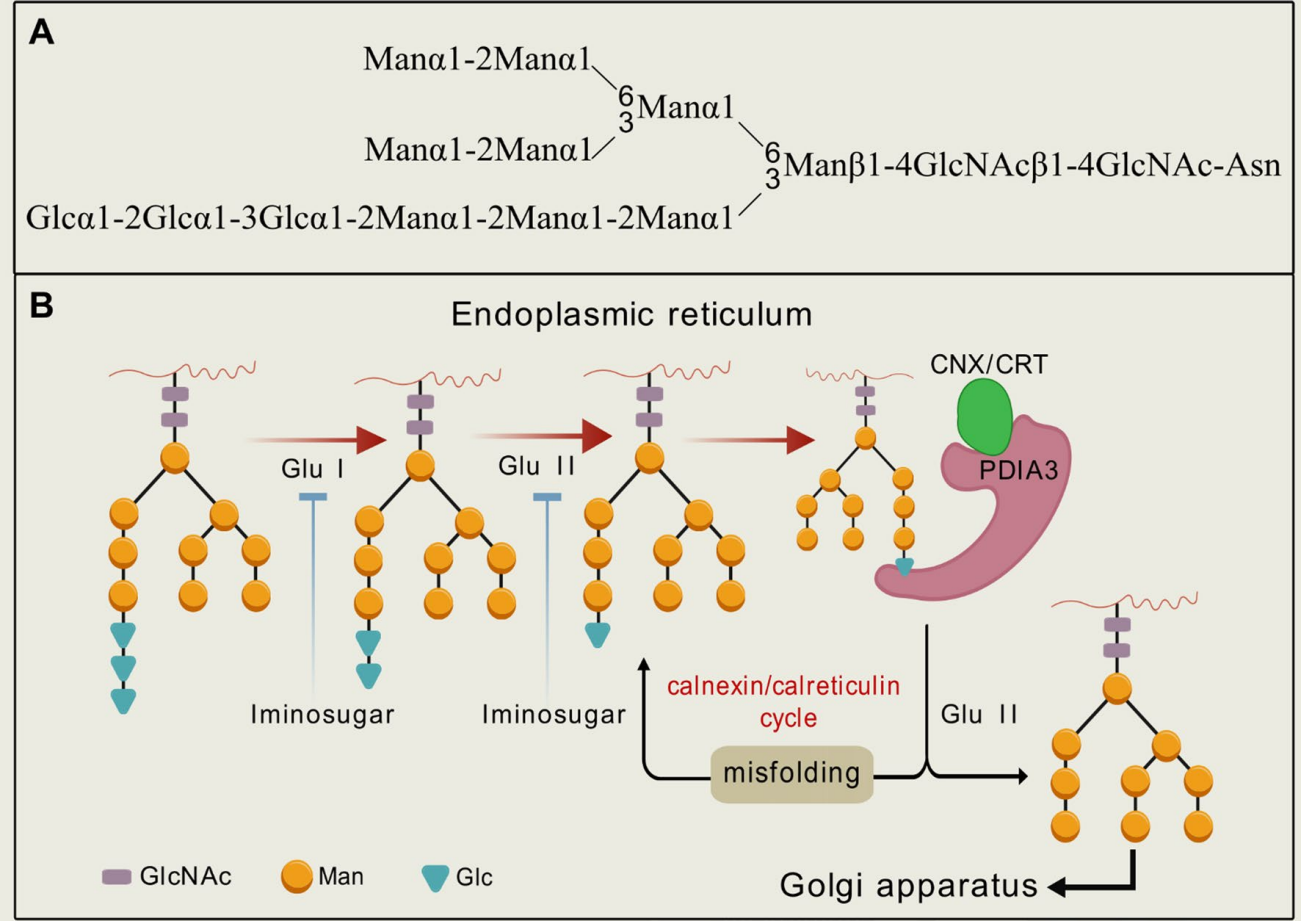
**A** Schematic representation of the precursor *N*-glycan Asn-Glc_3_Man_9_GlcNAc_2_. **B** The *N*-glycan processing of glycoproteins in ER

Due to their basic nature, iminosugars are protonated under the physiological pH, forming the ammonium ions that mimic the charge and shape of the anomeric carbocation or oxocarbenium-ion transition state involving in the glycoside hydrolysis to inhibit ER α-glucosidases I and II [27,28], [29] This leads to the accumulation of unprocessed viral glycoproteins and disrupts their interactions with calnexin, thereby impeding viral glycoprotein folding (Fig. 1). For instance, iminosugars have been shown to interfere with various viral glycoproteins, including the membrane-associated E-proteins of flavivirus [30,31], the spike proteins of coronavirus [32] and the gp120 and gp41 of HIV-1 [33,34]. In addition, iminosugars also target host glycoprotein angiotensin converting enzyme 2 (ACE2) to disrupt viral entry [35,36].

### Non-α-glucosidase inhibitory mechanisms

There are inconsistence between the antiviral activity of iminosugars and their inhibition of ER α-glucosidases, suggesting the additional mechanisms contributing to their antiviral efficacy. For instance, some iminosugars, such as *N*-nonyl-1-deoxynojirimycin (NN-DNJ) and 1-deoxygalactonojirimycin (DGJ) derivatives, retain their antiviral effects despite diminished glucosidase inhibitory activity, indicating multifaceted modes of actions.

Recent studies have revealed that iminosugars can directly interact with viroporins. For instance, HBx, a multifunctional non-structural protein of HBV, orchestrates membrane permeability and modulates intracellular conditions to facilitate viral production. NN-DNJ significantly inhibited HBx-induced mitochondrial depolarization in HBV [37] by disrupting the aggregation or formation of HBV virion particles through direct interactions with HBx proteins, thereby impeding HBV encapsulation [38]. Similarly, the DGJ derivatives [39], *N*-butyl-1-deoxynojirimycin (NB-DNJ) [40], and NN-DNJ [41] disrupted p7 ion channel oligomerization of Hepatitis C viruses (HCV) by binding to the interface of the p7 monomers, thereby interfering viral particle assembly and release [41]. NN-DNJ primarily binds to the protomer interface of the p7 protein monomer rather than the entire channel complex. The F25A mutation in the GT3a subtype of HCV significantly impacts the binding capacity of p7 to NN-DNJ, rendering it resistant to NN-DNJ both in vitro and in vivo*.* Additionally, NB-DNJ has been shown to inhibit HIV activity in vitro [42] by reducing their binding to CD4 + T cells [43]. Mechanistically, NB-DNJ changes the glycan composition of HIV gp41 protein 22 [33], affecting the conformation of the V1/V2 loops and the overall charge of the C1 and C2 regions of the HIV gp120 protein [44]. This results in a four-fold reduction in the exposure of virion-associated gp41 epitopes [33]. Such binding to gp120 has also been observed with *N*-stearyl-DNJ [45]. Besides, molecular simulations and dynamics studies revealed that DNJ derivatives effectively interact with the active site of SARS-CoV-2 main proteases (M^pro^), a critical protease responsible for cleaving neo-coronavirus proteins essential for viral replications [16].

Moreover, iminosugars have been developed as selective inhibitors of glycolipid biosynthesis. For instance, NB-DNJ, also known as Miglustat (Zavesca), inhibits glucosylceramide synthase (GCS) by mimicking the substrate ceramide [46]. Intracellular glycosphingolipids (GSLs), analogous to iminosugars, are initially synthesized with the catalysis of GCS to form glucosylceramide (GlcCer) as the intermediate. Some enveloped RNA viruses rely on host lipids for replication, which underscores the intricate interplays between GSLs and viral replication processes [45,47–49]. Inhibition of GlcCer synthesis has been shown to significantly reduce Zika virus (ZIKV) titers [50]. GCS inhibitors have also been shown to suppress the infections and replications of SARS-CoV, SARS-CoV-2 and influenza viruses, thereby reducing mortality and accelerating viral clearance [51,52]. Furthermore, several iminosugars with specific sugar cores (i.e., furanoses and pyranoses) have been explored as potent protease inhibitors against HIV [53]. Notably, there is currently no direct evidence suggesting that iminosugars exert antiviral effects by interfering lipid metabolism processes.

## Structure biology studies

Given their remarkable antiviral effects and unique modes of action, iminosugars have garnered significant attentions from structural biology community. Investigations have provided insights into how factors, such as ring size, types of *N*-substituents, and stereochemistry, influence the inhibition and specificity of mammalian α-glycosidases [54]. These studies offer a detailed understanding of the molecular interactions that underpin the antiviral properties of iminosugars, facilitating the design of more effective antiviral agents.

### The binding between iminosugars and ER α-glucosidase I

**Fig. 2:**
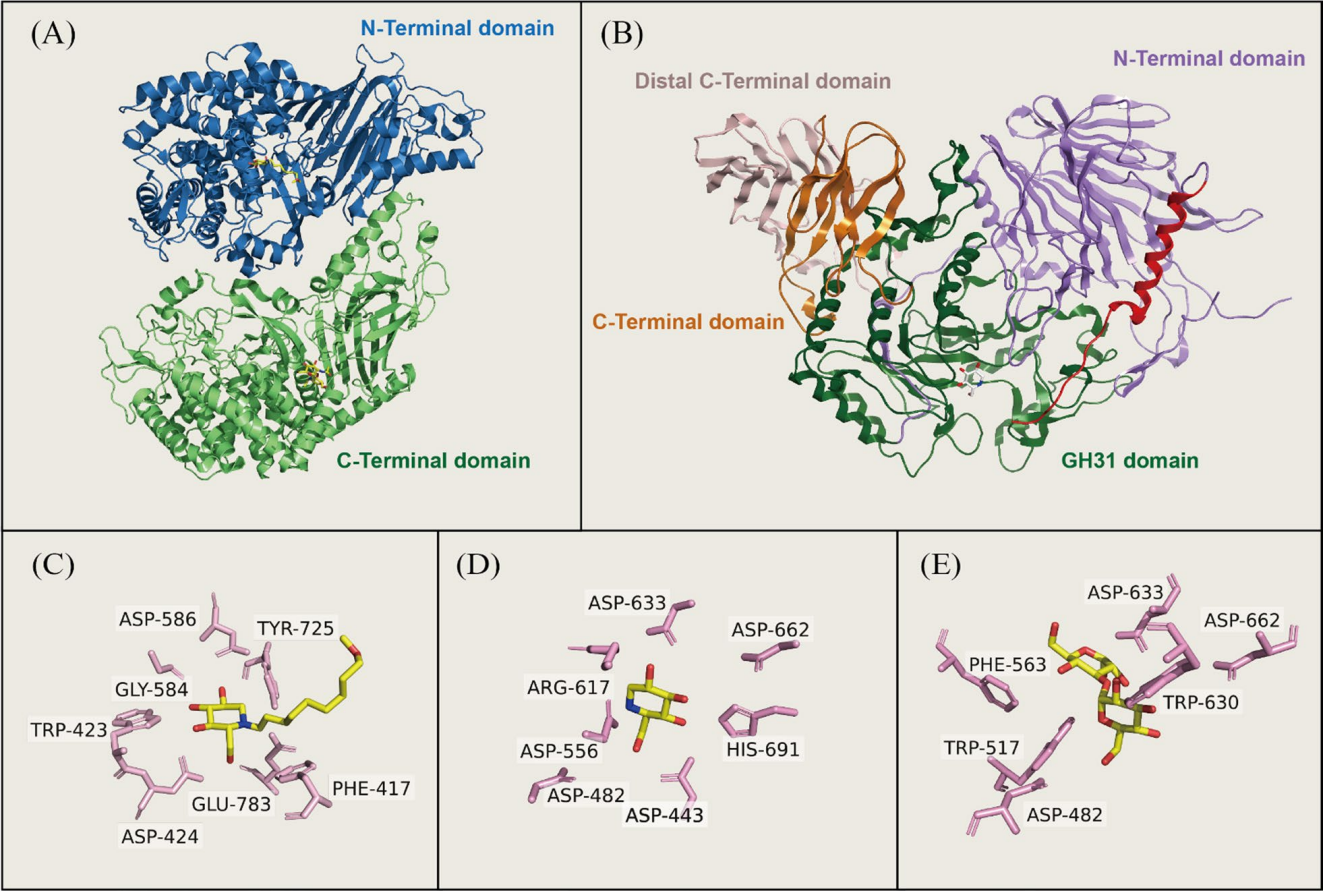
**A** The ribbon diagram illustration of the crystal structure of α-glucosidase I from *Thermochaetoides thermophila* (PDB entry: 7T6W); **B** The ribbon diagram illustration of the crystal structure of α-glucosidase II α-subunit from *Thermochaetoides thermophila* (PDB entry: 5DKX); **C** Complexe structure of *Thermochaetoides thermophila*-derived α-glucosidase I with MON-DNJ (PDB entry: 7T66); **D** Complexe structure of *Thermochaetoides thermophila* -derived α-glucosidase II with DNJ (PDB entry: 5DKY); **E** Complexe structure of *Thermochaetoides thermophila*-derived α-glucosidase II with Glc-α1,3-Glc (PDB entry: 5DKZ)

Eukaryotic α-glucosidase I, also known as mannosyl-oligosaccharide glucosidase (EC 3.2.1.106), is a transmembrane glycoprotein classified under Glycoside Hydrolase (GH) 63 family. It consists of an *N*-terminal domain and a *C*-terminal catalytic domain (Fig. 2A). The *C*-terminal catalytic domain is highly conserved across the species, featuring an (α/α) 6-ring bundle at the active center [55]. Species differences manifest as slight variations in some aromatic residues, including Phe416, Phe417, Phe475, and Tyr725. Additionally, the disulfide bond between Cys654 and Cys668 is observed across some species (i.e., *Saccharomyces cerevisiae* α-glucosidase I) but absent in others (i.e., *Mus musculus* α-glucosidase I), potentially contributing to the enhanced thermostability of certain variants, such as *Chaetomium thermophilum* α-glucosidase I [29].

Presumably, Asp586 serves as the catalytic nucleophilic reagent, while Glu783 as the putative acid/base catalyst. As a result of the side chains of the iminosugars, the conformation of the enzyme changes in order to maximize binding to the sugar core. In particular, as the enzyme shifts to the open state, Asp586 directs residue toward the sugar core by rotation. Following substrate binding, Asp586 undergo rotational transformations (closed state) that enable Asp586 to act as the catalytic nucleophile. After catalysis, Asp586 return to open-state conformations, which prepares α-glucosidase I for a new catalytic cycle. Although different iminosugars have different side chains, their sugar cores are all bound to the same site in the α-glucosidase I. The hydroxyl groups of the sugar core form eight conserved hydrogen bonds with residues Trp423, Asp424, Gly584, Asp586, and Trp726 (Fig. 2C). Therefore, designing compounds against these residues mentioned above can effectively increase their inhibitory activity.

### The binding between iminosugars and ER α-glucosidase II

ER α-glucosidase II is a heterodimer composed of a catalytic α-subunit and an accessory β-subunit, and its α-subunit comprises four structural domains (Fig. 2B). The α-glucosidase II belongs to the GH31 family of glycosyl hydrolases, which also comprises intestinal maltase-glucoamylase (MGAM) and sucrase-isomaltase (SI). While it shares a common catalytic mechanism with other GH31 α-glucosidases, ER α-glucosidase II uniquely exhibits α-1,3-glucosidase activity.

ER α-glucosidase II cleaves the second and third glucoses of the precursor Glc2Man9GlcNAc2 in a similar manner. The second α-1,3-linked glucose residue, which closely resembles that of DNJ, occupies the deep subsite, while the third α-1,3-linked glucose occupies a surface subsite. Kinetic studies suggest that the two-step glucose trimming reaction catalyzed in the ER α-glucosidase II is discontinuous due to its gourd-shaped structure. The first cleavage step is more efficient than the second. The initial glucose product needs to be removed from the deep subsite, and then pass through the surface subsite before the remaining structure can accommodate by the substrate's second cleavage site. Thus, the first glycosidic hydrolysis step is crucial for the antiviral activity [56,57].

Hydrophobic residues Asp564 and Asp640, which are nucleophilic and general acid–base catalysts, sandwich the substrate. ER α-glucosidase II selectivity determinants are located outside the catalytic pocket. Hydrophobic residues Phe307 and Gln308 that are specific in α-glucosidase II block the insertion of acarbose, thus determine its selectivity. DNJ hydroxyl portion interacts with α-glucosidase II similarly to Glc-α1,3-Glc, and its endocyclic nitrogen atom is in close proximity to the catalytic Asp564 (Fig. 2D, E). Designing compounds for these hydrophobic residues can improve their potency and selectivity for binding to enzymes [58].

Extensive evidence has established the structural basis of iminosugars’ inhibition against α-glucosidases and providing compelling insights into their impacts on viral glycoprotein processes. The different iminosugars are all equally bound to the catalytic structural domains of ER α-glucosidases via the glycan core, implying that the presence of free hydroxyl groups in the glycan core is essential for binding to ER α-glucosidases. Therefore, structural modifications of this part are very restricted and may inadvertently prevent the compound from binding to ER α-glucosidases Interestingly, DNJ and castanospermine (CAST) have very different structures, but both bind to the same subsite of α-glucosidase I. Furthermore, modifications to the N1-side chain can improve the lipophilicity. And, within certain limits, increasing the chain length can increase the inhibitory activity of the compound.

## Iminosugars as broad-spectrum anti-virals: their structure–activity relationships

Generally, antiviral iminosugars can be categorized into three distinct classes based on their core structures, namely the piperidine-type, pyrrolidine-type, and polycyclic iminosugars. This section will delve into their discovery, development, and the structure–activity relationships (SARs) of these iminosugars in the context of their antiviral properties.

### The piperidine-type iminosugars

The piperidine-type iminosugars, characterized by a six-membered ring, have shown significant antiviral activity against various viruses. Their structures allow for versatile modifications that can enhance binding affinity and specificity to target enzymes or proteins, leading to potent antiviral effects. Among these, DNJ and its derivatives are the most extensively studied for the antiviral therapy. In 1966, Inouye et al. first isolated nojirimycin (NJ, **1**) from *Streptomyces* [59,60]. However, NJ’s hemiaminal moiety renders it unstable under both alkaline and neutral conditions, undergoing elimination to form biologically inactive derivatives. Soon after, 1-deoxynojirimycin (DNJ, **2**) was isolated from the roots of *Morus alba*, which lacks an anomeric hydroxy group, thus conferring greater stability [61]. Since Bayer chemists first identified DNJ as a nonselective α-glucosidase inhibitor for the treatment of type 2 diabetes in 1979, DNJ and its derivatives have been renowned for their diverse pharmacological activities and promising therapeutic potentials [62]. Taylor and co-workers first disclosed that DNJ exerted its antiviral effect by blocking the Human cytomegalovirus (CMV) infections [63]. Subsequently, DNJ has demonstrated broad-spectrum antiviral effects, including bovine viral diarrhea virus (BVDV), HIV, and HCV.

**Fig. 3:**
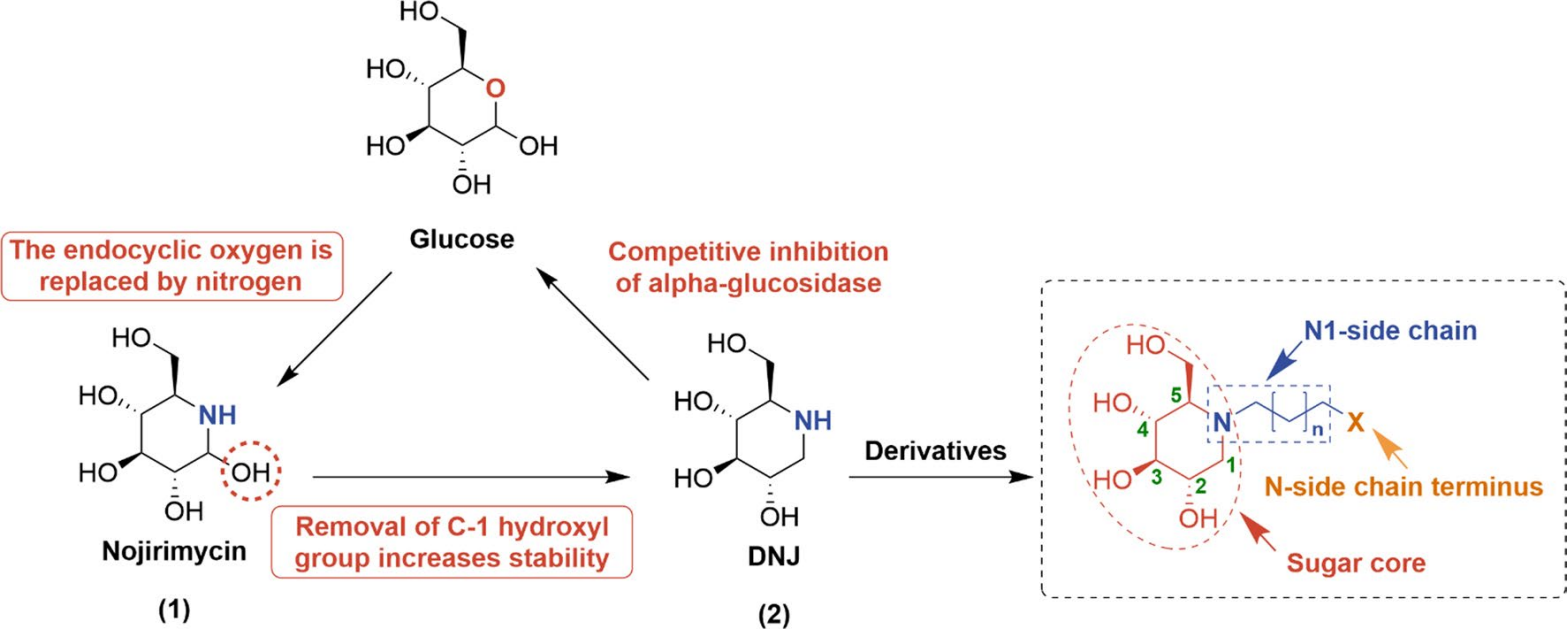
Chemical modifications of DNJ

Given its intriguing biological activity and unique modes of actions, extensive efforts have been directed towards exploring SARs of DNJ derivatives for their therapeutic potentials as novel antiviral agents. Generally, the chemical modifications of DNJ primarily involve the alterations to the sugar core and *N*-side chain, typically by varying the length and composition of the *N*-side chain, as well as the substituents at the side chain terminus (Fig. 3).

#### DNJ derivatives with *N*-alkyl side chain

**Fig. 4:**
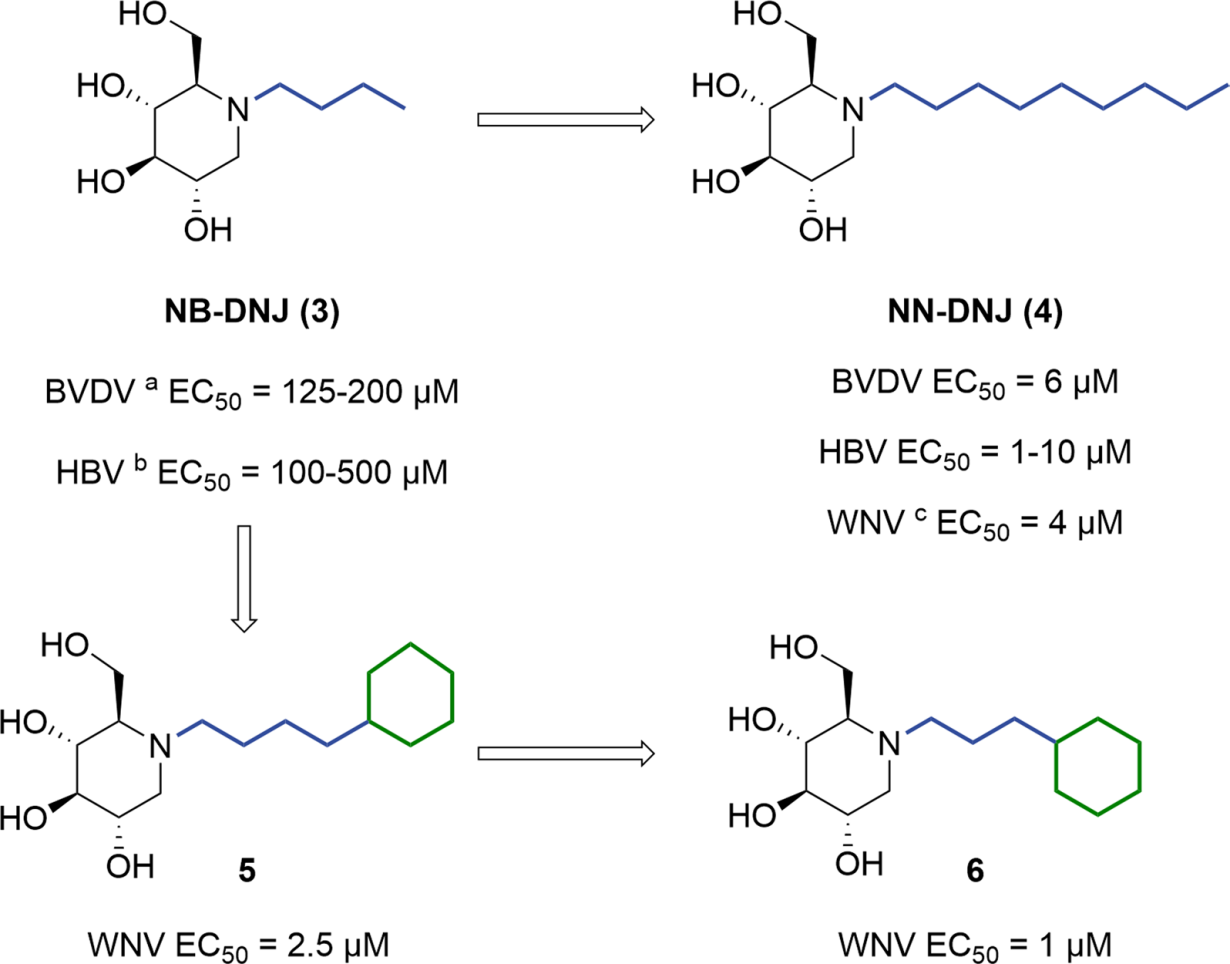
DNJ derivatives with *N*-alkyl side chains

DNJ derivatives with an *N*-alkyl side chain (Fig. 4) show widely varied antiviral efficacy, largely depending on the length of the *N*-alkyl side chain. Among these, derivatives with 4- and 9-carbon* N*-alkyl chains have shown notable antiviral efficacy (i.e., **3** and **4**). For instance, *N*-butyl-1-deoxynojirimycin (NB-DNJ, **3**) exhibited potent anti-HIV activity, particularly against the British HIV-1 isolate GB8 (EC_50_ = 56 μM, JM cells), which was nearly a ten-fold more potent than the parent compound DNJ [64]. In contrast, NN-DNJ (**4**) containing 9-carbon *N*-alkyl side chain was more active against BVDV and HBV viruses compared to NB-DNJ (i.e., **3** *vs*** 4**) [65,66]. In addition, NN-DNJ also exhibited remarkable inhibitory activity against three influenza A virus (IAV) of the H3N1, H3N2, and H1N1 subtypes in an HA-dependent manner, indicating its potentials to inhibit virus replications and transmissions [67], which was ten-fold more potent than NB-DNJ. Moreover, some *N*-alkyl DNJs, with a cyclohexyl terminal group on the side chain, also boosted potency against BVDV, and West Nile virus (WNV) (i.e., **5** and **6** *vs*** 4**) [22]. Notably, **6** demonstrated anti-HBV potency with EC_50_ of 30 μM in HepG2.2.15 cells by reducing the secretion of MHBs, an HBV envelope glycoprotein, to impede HBV replications [22].

*Generally,* *N*-alkylation of DNJ represents a straightforward and effective strategy for structural optimizations. Increasing the length of *N*-alkyl side chain or incorporation of a cyclohexyl terminal significantly enhances antiviral efficacy, although it also increases cytotoxicity. For instance, NN-DNJ, **5** and **6** (CC_50_ = 150–300 μM) showed nearly a tenfold increase in cytotoxicity compared to NB-DNJ (CC_50_ > 5000 μM). Notably, a consistent observation with *N*-alkyl DNJ is that a side chain length of 8–9 carbons or a cLogP of ~ 2.8–3.0 seems to be the optimal compromise between antiviral efficacy and acceptable cytotoxicity, which was called the “rule of nines”.

- The anti-BVDV activities were measured by yield reduction assay in Madin-Darby bovine kidney (MDBK) cells.
- Detected the HBV DNA levels in HepG2.2.15 cells by quantification by real time polymerase chain reactions (q-PCR).
- The anti-WNV activities were measured by yield reduction assay in baby hamster kidney (BHK) cells.

#### DNJ derivatives with* N*-alkyl side chain tethering an oxygen-containing substituent at the terminus

**Fig. 5:**
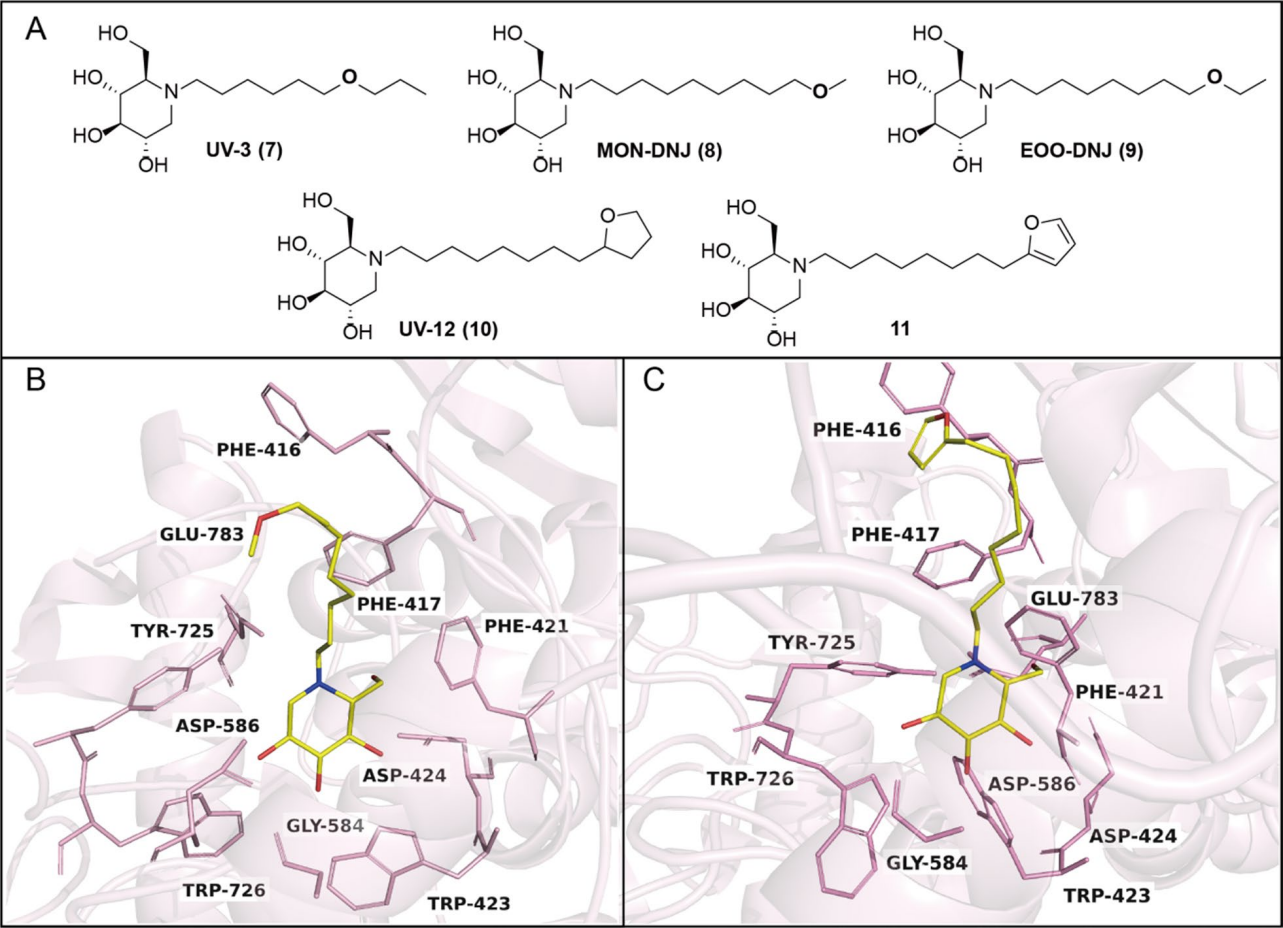
**A** Incorporation of oxygen-containing substituents at the *N*-side chain. **B** Complexes structure of Thermochaetoides thermophila-derived α-glucosidase I with MON-DNJ (PDB entry: 7T66). **C** Complexes structure of Thermochaetoides thermophila-derived α-glucosidase I with **11** (PDB entry: 8EUT)

Interestingly, incorporating an oxygen containing substituent into the *N*-side chain terminus of DNJ derivatives has shown promise in enhancing antiviral activity while reducing cytotoxicity. For instance, MON-DNJ (**8**, Fig. 5) exhibited low-micromolar activity against all four DENV serotypes in a virus yield reduction assay in Vero cells [68]. It also reversed DENV-mediated down-regulation of total IFNγ receptor expressions [69], suppressing virus-induced oxidative stress and pro-inflammatory cytokine productions [70]. Besides, the position of oxygen within the *N*-side chain significantly impacted the potency against DENV-2. For instance, *N*-oxadecyl-DNJ (UV-3, **7**) showed lower activity against DENV-2 compared to MON-DNJ [71], whereas compound **9** was more potent (EC_50_ = 3.1 μM). When the ethyl ether group in **9** was replaced by tetrahydrofuran (i.e., **10**), the potency increased further (EC_50_ = 1.6 μM). Both compounds **9** and **10** inhibited α-glucosidase I in DENV-infected imDCs and combated DENV infection by attenuating oxidative stress and reducing the virus-induced production of pro-inflammatory cytokines [6]. Similarly, when the *N*-alkyl side chain was incorporated with a furan ring (i.e., **11**), it increased the inhibitory activity of α-glucosidase I by four folds. From the complexes structure of ER α-glucosidase I with the iminosugars (Fig. 5B and C, which resulted in increased in vitro glycosidase inhibitory activity [72]. Unfortunately, it did not improve their anti-SARS-CoV-2 activity.

**Fig. 6:**
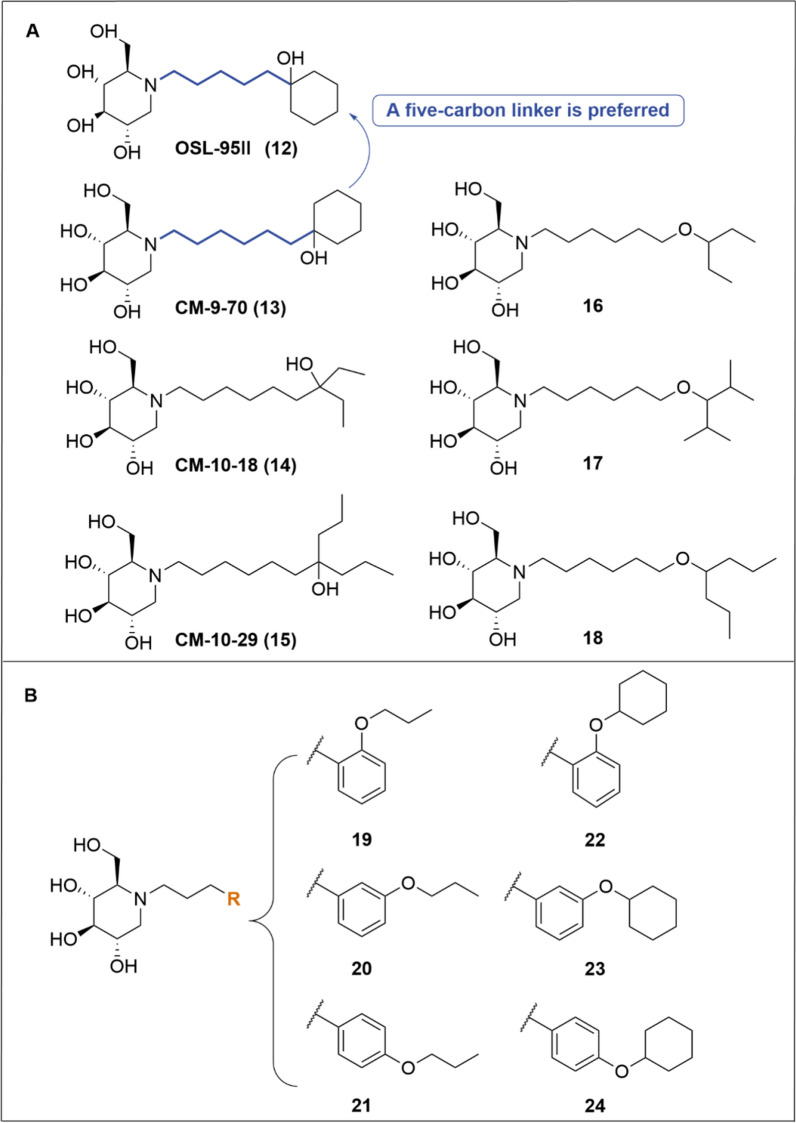
**A** DNJ derivatives with a hydroxyl cyclohexyl group at the* N*-side chain terminus. **B** DNJ oxygen-containing derivatives with 3-carbon *N*-side chain

For the anti-BVDV activity, introducing a tertiary alcohol group at the *N*-side chain terminus (i.e., **12–15**, Fig. 6A) maintained potency in MDBK cells compared to MON-DNJ (Table 1). Wherein, the 5-carbons linker was more favorable than the 6-carbons linker (i.e., **12** *vs* **13**). Acyclic *di*-alkyl substituents at the terminus also demonstrated widely varied inhibitory activity against BVDV. For instance, compound **15** with the *bis*-ethyl terminal group (EC_50_ = 9.5 μM) was two-fold more potent than that of **14** with the *bis*-propyl group (EC_50_ = 22 μM) [73]. Changing the tertiary alcohol group of **14** and **15** into the corresponding ether led to enhanced anti-BVDV activity and no observable cytotoxicity (i.e., **14** *vs* **16**, **15** *vs ***18**.) [74].

**Table 1:** The anti-BVDV activities of **9, 12–24** in MDBK cells

Entry	EC_50_^a^ μM)	CC_50_ (µM)	References	Entry	EC_50_ (μM)	CC_50_ (µM)	References
**9**	7.3 ± 7.1	245 ± 134	[73]	**18**	0.6	> 500	[74]
**12**	10.5 ± 2.7	> 500	[73]	**19**	0.8	> 500	[74]
**13**	18.0	> 500	[73]	**20**	1.2	> 500	[74]
**14**	22.0 ± 8.5	> 500	[73]	**21**	0.2	> 500	[74]
**15**	9.5 ± 0.7	> 500	[73]	**22**	24.0	420	[74]
**16**	3.0	450	[74]	**23**	3.5	390	[74]
**17**	7.5	> 500	[74]	**24**	5.5	295	[74]

^a^The anti-BVDV activities were measured by yield reduction assay in MDBK cells

CC_50_, 50% cytotoxic concentration; EC_50_, half maximal effective concentration

Besides, oxygen-containing aryls at the *N*-side chain terminus (i.e., **19–24**, Fig. 6B) also exerted potent anti-BVDV activities and low cytotoxicity (Table 1). The substituents on the aryl group exerted substantial impacts on the efficacy. Notably, introducing bulky groups at the aryl moiety were not favorable. For instance, the cyclohexyl ether on the aryl moiety exhibited decreased antiviral activity compared to the corresponding propyl ether congeners (i.e., **19** *vs* **22**, **20** *vs* **23**, **21 ***vs* **24**). Besides, the position of propoxy ether exerted a negligible impact on the anti-BVDV activity, whereas the cyclohexyl ether at the *ortho*-position led to a significant decreased activity (i.e., **22** *vs* **23** and **24**).

**Fig. 7:**
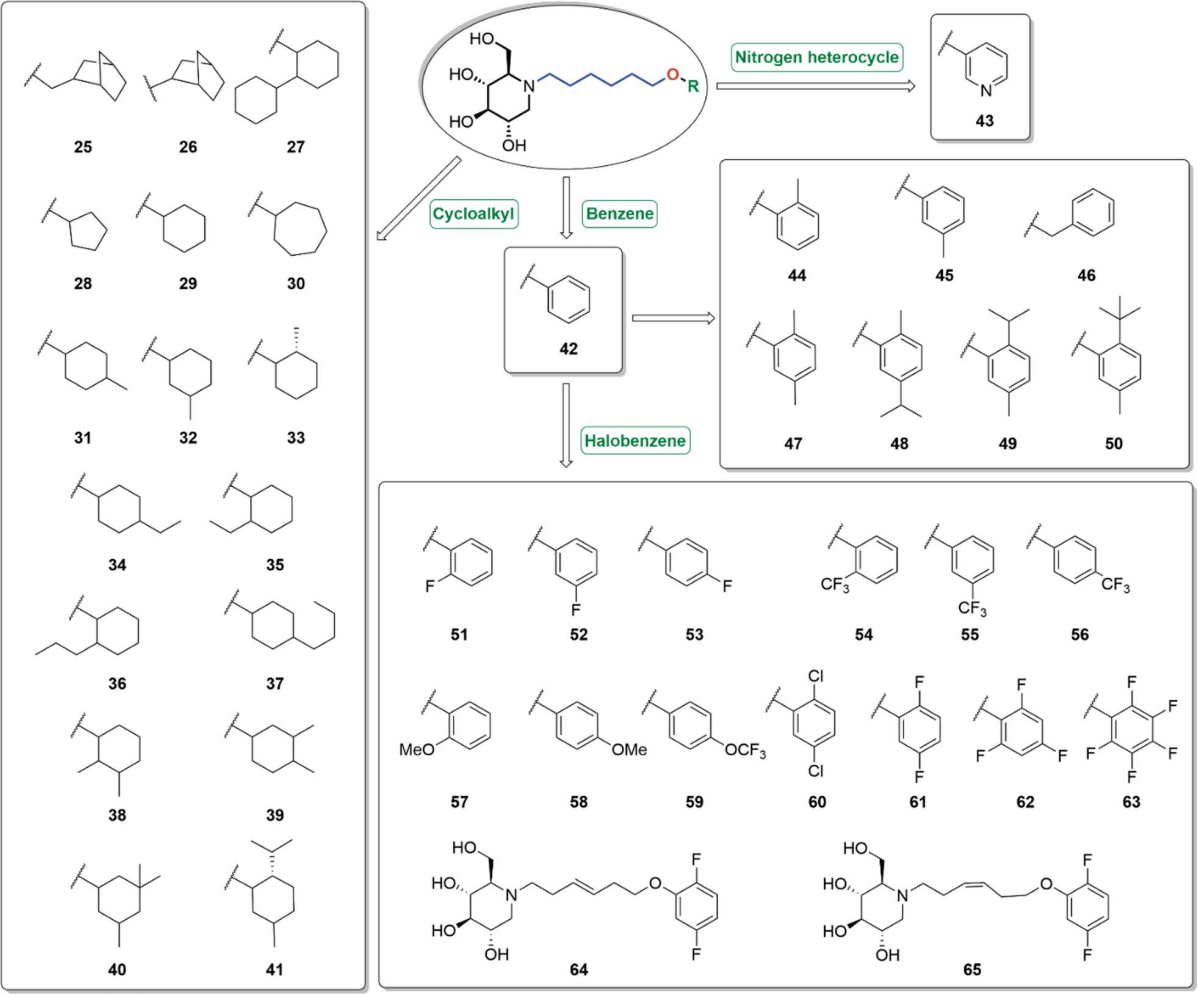
Representative DNJ derivatives with oxygen-containing groups at the *N*-side chain terminus

The alkyl and aryl ether group at the side chain terminus significantly influenced the anti-BVDV efficacy and cytotoxicity (i.e., **25–65**, Fig. 7 and Table 2) [74]. The bridged cyclic ether groups resulted in a decreased activity (i.e., **25** and **26**), while the dicyclohexylidene maintained the potency (i.e., **27**). The size of substituent at the side chain terminus also affected the anti-BVDV potency. The cyclopentyl (i.e., **28**, EC_50_ = 0.3 μM) and cyclohexyl (**29**, EC_50_ = 0.4 μM) ethers were optimal, while the cycloheptyl ether (i.e., **30**, EC_50_ = 1.4 μM) showed slightly reduced activity. Additional alkyl groups on the cyclohexane ring substantially impacted the activity. Compared to **29**, the *meso*-methyl cyclohexane remained active (i.e., **32**), while the *ortho*- and *para*-methyl substituents reduced the activity by more than 10 folds (i.e., **31** and **33**) [58]. The steric effects were also investigated. Generally, substituents at the *meta*- and *para*- positions (i.e., **32** and **34**) exhibited more potent anti-BVDV profiles compared to the *ortho*-counterpart (i.e., **35**). Derivatives with multiple or bulky substituents on the terminal cyclohexyl ring displayed decreased activity and/or increased cytotoxicity (i.e., **36–41**).

**Table 2:** The anti-BVDV and DENV-2 activities of DNJ derivatives **4**, **14**, **25**–**65**

Entry	BVDV^a^ (MDBK cell)		DENV-2^b^ (BHK cell)		References	Entry	BVDV (MDBK cell)		DENV-2 (BHK cell)		References
	EC_50_	CC_50_	EC_50_	CC_50_			EC_50_	CC_50_	EC_50_	CC_50_	
**4**	1.1	350	ND^c^	ND	[74]	**44**	0.4	> 500	0.5	450	[74]
**14**	6.2	> 500	6.5	> 500	[74]	**45**	0.6	> 500	0.6	> 500	[74]
**25**	2.9	> 500	ND	ND	[58]	**46**	1.5	500	0.4	> 500	[74]
**26**	4.5	> 500	ND	ND	[58]	**48**	0.7	90	ND	ND	[74]
**27**	0.5	200	ND	ND	[74]	**49**	0.6	62	ND	ND	[74]
**28**	0.3	> 500	1.4	> 500	[74]	**50**	0.6	85	ND	ND	[74]
**29**	0.4	480	1.6	> 500	[74]	**51**	2.8	> 500	ND	ND	[74]
**30**	1.4	> 500	0.5	> 500	[74]	**52**	0.5	> 500	0.4	> 500	[74]
**31**	7.4	53	6.75	> 40	[58]	**53**	0.3	> 500	1.1	> 500	[74]
**32**	0.3	> 500	ND	ND	[74]	**54**	1.0	> 500	ND	ND	[74]
**33**	3.3	> 500	0.075	65	[58]	**55**	1.5	> 500	0.4	400	[74]
**34**	0.4	> 500	0.3	475	[74]	**56**	1.1	> 500	1.3	470	[74]
**35**	1.5	450	ND	ND	[74]	**57**	1.0	> 500	0.8	> 500	[74]
**36**	0.5	150	ND	ND	[74]	**58**	1.0	> 500	4.0	> 500	[74]
**37**	1.8	190	ND	ND	[74]	**59**	1.3	> 500	0.6	> 500	[74]
**38**	1.2	> 500	ND	ND	[74]	**60**	1.5	320	ND	ND	[74]
**39**	3.5	> 500	0.4	> 500	[74]	**61**	0.4	> 500	ND	ND	[74]
**40**	0.8	250	ND	ND	[74]	**62**	5.0	> 500	ND	ND	[74]
**41**	ND	ND	0.1	> 500	[74]	**63**	1.0	> 500	1.7	> 500	[74]
**42**	0.9	> 500	1.7	> 500	[74]	**64**	0.9	> 500	2.2	> 500	[74]
**43**	2.5	> 500	ND	ND	[74]	**65**	2.0	> 500	8.5	> 500	[74]

^a^The anti-BVDV activities were measured by yield reduction assay in MDBK cells

^b^The anti-DENV-2 activities were measured by yield reduction assay in BHK cells

^c^ND, not determined

CC_50_, 50% cytotoxic concentration; EC_50_, half maximal effective concentration

Additionally, aryl group (i.e., **42**) was slightly less effective than the corresponding cyclohexane counterpart (i.e., **29**), but more effective than the pyridine group (i.e., **43**). Alkyl substituents on the aryl ring also slightly increased the activity, whereas the short-chain alkyl groups at different sites exerted negligible impacts on the activity (i.e., **44–50**). Notably, compounds with bulky substituents (i.e., **48–50**) displayed significantly cytotoxicity.

Interestingly, derivatives with *mono*- or *di*-fluorine (i.e., **52**, **53** and **61**) at the terminal ring maintained the good anti-BVDV activity. Replacing the fluorine with chloro-group produced a similar effect (i.e., **60** and **61**). Derivatives with fluorine atom at *meta-* and *para-* position (i.e., **52** and **53**) exerted better activity than those with *ortho-*fluorine atom (i.e., **51**). Even pentafluoro-groups (i.e., **63**) exhibited moderately improved activity, while with no observable cytotoxicity. This position preference was not observed in other aryl substituents, such as trifluoromethyl (i.e., **54–56**), methoxy (i.e., **57** and **58**), and trifluoromethoxy (i.e., **59**) groups.

Notably, these derivatives not only demonstrated excellent anti-BVDV activity, but also exhibited remarkable potency against DENV [73]. In contrast to their anti-BVDV activity, **30** with a larger terminal ring possessed threefold more potent anti-DENV activity than compounds **28** and **29**. *Ortho-*methyl group on the terminal benzene ring (i.e., **31**–**33**) also exerted more potent activity and lower cytotoxicity. Besides, the anti-DENV activity of cyclohexane (i.e., **29**) was comparable to that of aromatic ring substitution (i.e., **42**). *Meta-*methyl group on the terminal benzene ring showed comparable activity to *ortho-*methyl group (i.e., **44** and **45**). Compound **46** with one extra carbon between the ether oxygen and the terminal ring also exhibited excellent activity, contrasting to the remarkable reduced activity against BVDV.

Introducing the *mono-* or *di-*fluorine (i.e., **52**, **53** and **63**) of the terminal ring maintained the good anti-DENV activity. Further analysis revealed that derivative with fluorine atom at *meta-*position (i.e., **52**) gave better activity than those with fluorine atom at *para-*position (i.e., **53**). Replacing the methoxy group in **53** with a trifluoromethoxy group (i.e., **59**) resulted in a more than sixfold improvement in activity. However, penta-fluorine (i.e., **63**) on the terminal benzene ring slightly decreased the activity. Derivatives with restrained linker (i.e., **64** and **65**) showed poor anti-DENV activity, confirming that a linker with six carbons is optimal.

#### DNJ derivatives with* N*-alkyl side chain tethering a nitrogen-containing substituent at the terminus

**Fig. 8:**
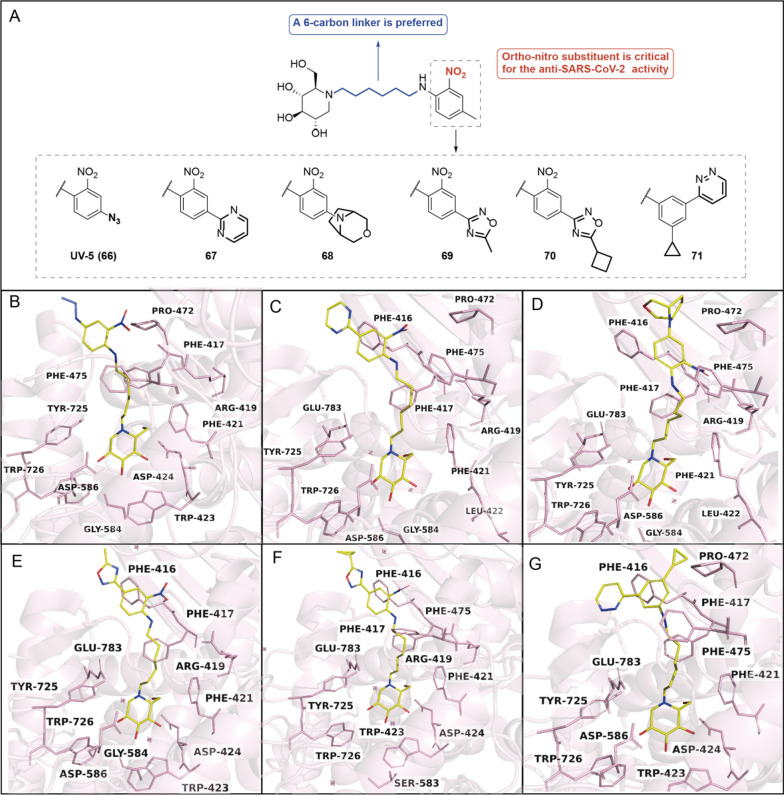
**A** DNJ derivatives with nitrogen-containing groups at the *N*-side chain terminus showing anti-SARS-CoV-2 activities; **B**–**G** Complexes structure of Thermochaetoides thermophila-derived α-glucosidase I with UV-5 (PDB entry:7T66), **67 **(PDB entry:8E3J), **68 **(PDB entry:8E5U), **69 **(PDB entry:8E4I), **70 **(PDB entry:8E4Z), **71 **(PDB entry:8E6G), respectively

As mentioned above, variations at *N*-side chain of DNJ derivatives demonstrate intriguing structure–activity relationship (SAR) profiles, particularly when introducing a nitrogen atom at the side chain terminus. DNJ derivatives with a nitrogen-containing substituent at the side chain terminus have been shown with anti-SARS-CoV-2 activities (i.e., **66–71**, Fig. 8A) [72]. Wherein, the composition of the linker was a determinant for the anti-SARS-CoV-2 activity. Derivatives with *para-*phenyl linkers (i.e., **66–70**, Table 3) were more effective than those with *meta-*phenyl linkers (i.e., **71**, Table 3). Structural biology studies revealed that these inhibitors extensive exerted interactions with all enzyme active site of α-glucosidase I (i.e., Fig. 8B–G). The primary aminophenyl ring formed π − π interactions with Phe417, and/or Phe475 in the different α-glucosidase I-inhibitor complexes. The π − π interactions with Phe416, Pro472 mediated by the secondary and tertiary rings, which were absent from UV-5, may explain the enhanced inhibitory potency of **67**–**70**.

**Table 3:** The anti-SARS-CoV-2 activities of **66–71** in ACE2-A549 cells ^72^

Entry	EC_50_ (μM)^a^	IC_50_ (μM)^b^	CC_50_ (μM)
66	3.36	0.155	35
67	0.42	0.057	> 100
68	1.61	0.052	> 100
69	0.44	0.099	> 100
70	0.72	0.011	41
71	7.86	0.103	> 100

^a^The anti-SARS-CoV-2viral activities were measured by yield reduction assay in ACE2-A549 cells

^b^The enzymatic inhibitory activity against purified recombinant α-glucosidase I was determined in a 96-well plate assay using the trisaccharide substrate and a glucose oxidase reporter system which allowed quantification of released α-d-glucose

**Fig. 9:**
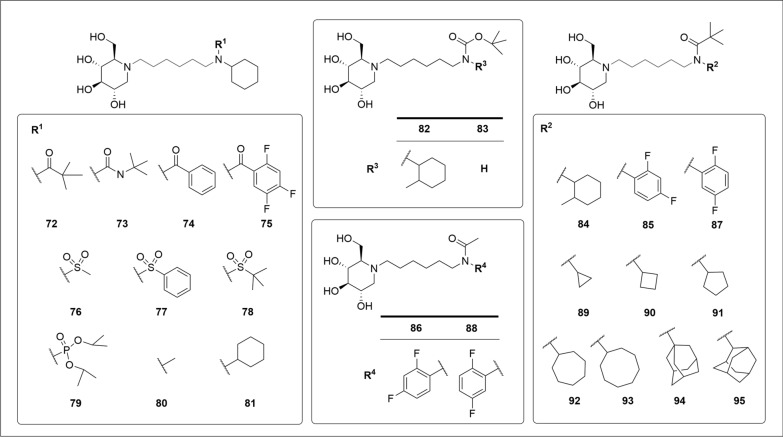
DNJ derivatives with nitrogen-containing groups at the* N*-side chain terminus showed anti-BVDV and anti-TARV activities

Similar to the oxygen counterpart, nitrogen-containing DNJ derivatives with six-carbon linkers exhibited good anti-BVDV activity (i.e., **72–95**, Table 4). Wherein, the R^1^ and R^2^ substituents can be varied (Fig. 9). For instance, DNJ derivative **72** with the *N*-pivaloyl and -cyclohexyl groups at the *N*-side chain terminus demonstrated excellent anti-BVDV activity (EC_50_ = 0.2 μM) [75,76]. Changing the R^1^ group to either phenyl (i.e., **74**, EC_50_ = 0.32 μM), 2,4,5-fluorophenyl (i.e., **75**, EC_50_ = 0.27 μM) or dicyclohexyl groups (i.e., **81**, EC_50_ = 0.28 μM) retained the antiviral potency and low cytotoxicity [77]. In contrast, the methyl group (i.e., **80**, EC_50_ = 6.3 μM), as well as urea group (i.e., **73**, EC_50_ = 3.0 μM), exhibited decreased efficacy. Replacing R^1^ carbonyl group with sulfonyl group resulted in a decreased activity, especially when substituted with a phosphoryl group (i.e., **79**, EC_50_ = 3.4 μM) [77].

**Table 4:** The anti-BVDV, and TARV activities of **72–95**

Entry	EC_50_ (μM)		CC_50_ (µM)	References
	BVDV^a^ (MDBK cell)	TARV^b^ (Huh7.5 cell)		
**72**	0.20	0.20	> 500	[77]
**73**	3.00	2.50	> 500	[75]
**74**	0.32	0.60	> 500	[77]
**75**	0.27	0.60	> 500	[77]
**76**	0.20	0.70	> 500	[77]
**77**	0.50	1.60	200	[77]
**78**	0.31	0.40	ND	[77]
**79**	3.40	0.30	310	[77]
**80**	0.28	ND^c^	250	[77]
**81**	6.30	ND	> 500	[76]
**82**	0.40	ND	250	[76]
**83**	2.50	ND	> 500	[76]
**84**	0.22	ND	> 500	[76]
**85**	0.25	ND	> 500	[76]
**86**	6.00	ND	> 500	[76]
**87**	0.40	ND	> 500	[76]
**88**	5.00	ND	> 500	[76]
**89**	3.75	ND	> 500	[76]
**90**	0.80	ND	> 500	[76]
**91**	0.85	4.00	> 500	[78]
**92**	0.40	1.00	> 500	[78]
**93**	0.24	0.29	150	[78]
**94**	0.31	0.50	125	[78]
**95**	0.21	0.21	170	[78]

^a^The anti-BVDV activities were measured by yield reduction assay in MDBK cells

^b^The anti-TARV activities were measured by yield reduction assay in Huh 7.5 cells

^c^ND, not determined

CC_50_, 50% cytotoxic concentration; EC_50_, half maximal effective concentration

For R^2^ group, various ring substituents were effective. Wherein, the aliphatic rings larger than four-membered rings (i.e., **90–94**) were more effective. Cyclopropyl group led a significant drop of the activity (i.e., **89**, EC_50_ = 3.75 μM). However, alkyl-substituted cyclohexanes (i.e., **84**, EC_50_ = 0.22 μM), fluorine-substituted aryl (i.e., **85** and **87**), or adamantanes (i.e., **94** and **95**) maintained good anti-BVDV activity [76].

Overall, it’s necessary to incorporate a ring substituent at *N*-terminus to achieve good anti-BVDV activities (i.e., **82** and **83**). Removing the *tert*-butyl (or lipophilic group) at the R^4^ position resulted in a more than 10-folds decrease in activity (i.e., **86** and **88**). Notably, **72–79** and **91–95** exhibited excellent anti-TARV activity (Table 4). Differences in R [1] substituents seemed to exert little impacts on the activity, while five-membered or larger aliphatic R^2^ ring substituents appeared to be beneficial (EC_50_ < 1 μM) [78].

#### Modifications at the sugar core

**Fig. 10:**
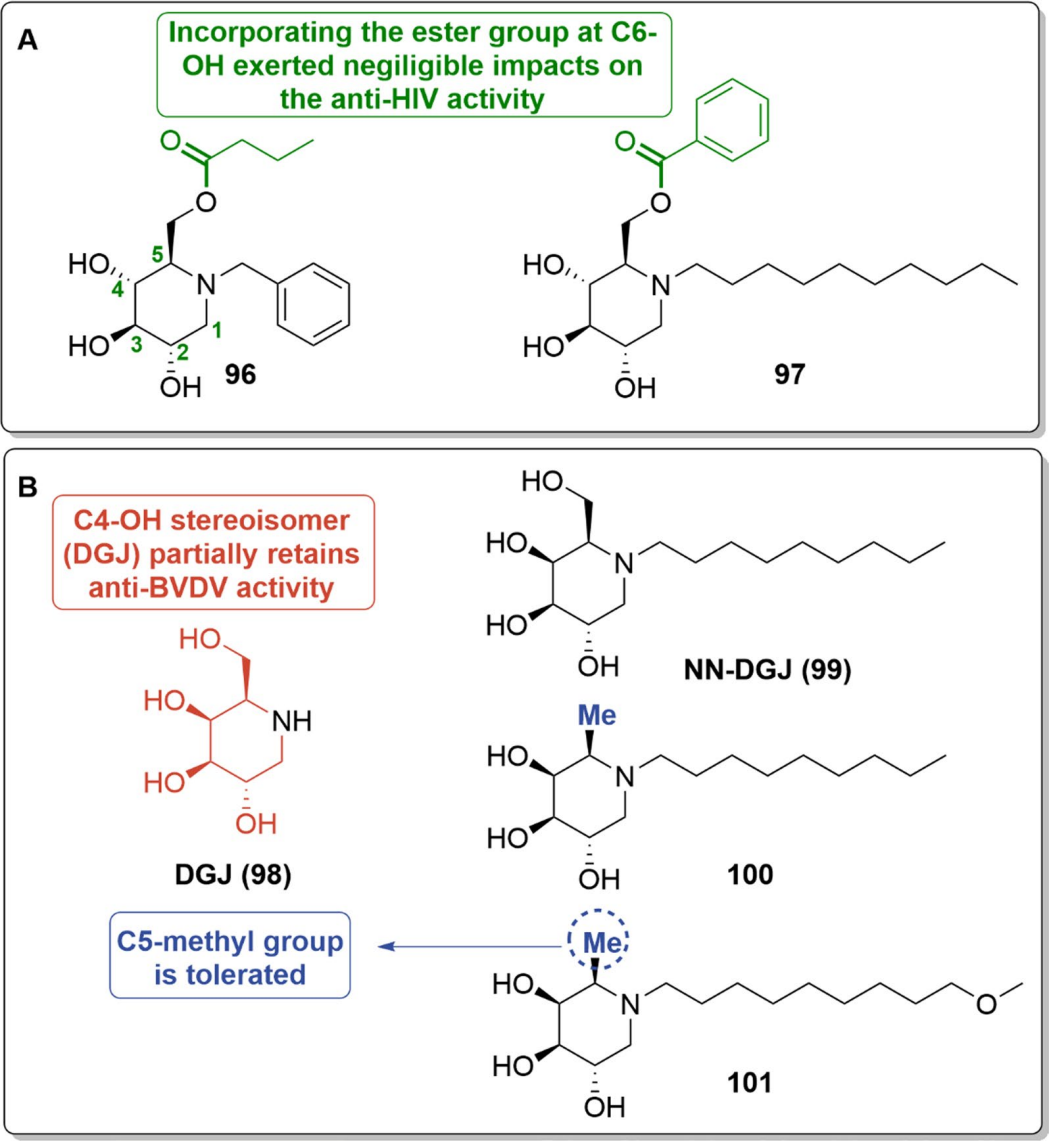
Chemical modifications at the sugar core: **A** The esterification at C6-OH; **B** Chemical modifications of DGJ

Modifications at the sugar core of iminosugars have been relatively limited in antiviral researches [79]. Subtle variations in the DNJ core often result in a reduced or complete loss of ER α-glucosidase inhibitory activity, thereby significantly impacting their antiviral efficacy [80,81]. Exceptionally, in an anti-HIV study, incorporating an ester group at C6-position of the sugar core did not significantly affect the activity compared to the parent compounds (i.e., **96–97**, Fig. 10A) [82].

Some variations of the sugar core gave the corresponding galactose isomers. As a typical example, alkovirs (i.e., **98–101**, Fig. 10B) represent a class of DGJ (Migalastat, **98**) derivatives [83]. Despite the absence of ER α-glycosidase inhibitory activity, alkovirs still exhibited remarkable antiviral activities. For instance, **99**, **100**, and **101** exhibited genotype-specific inhibitory activity against BVDV at a low viral titer (MOI = 0.01) (Table 5) [66].

**Table 5:** The anti-BVDV activities of **99**, **100**, **101** in MDBK cells ^66^

Entry	EC_50_ (μM)^a^			CC_50_ (µM)
	MOI = 0.01	MOI = 0.1	MOI = 1	
**99**	2.5 ± 0.5	7.5 ± 2.5	25 ± 5	237.5 ± 12.5
**100**	2.0 ± 0.5	6.5 ± 1.5	25 ± 5	187.5 ± 12.5
**101**	2.5 ± 0.5	17.5 ± 2.5	125 ± 25	> 4000

^a^The anti-BVDV activities were measured by yield reduction assay in MDBK cells

### The pyrrolidine-type iminosugars

**Fig. 11:**
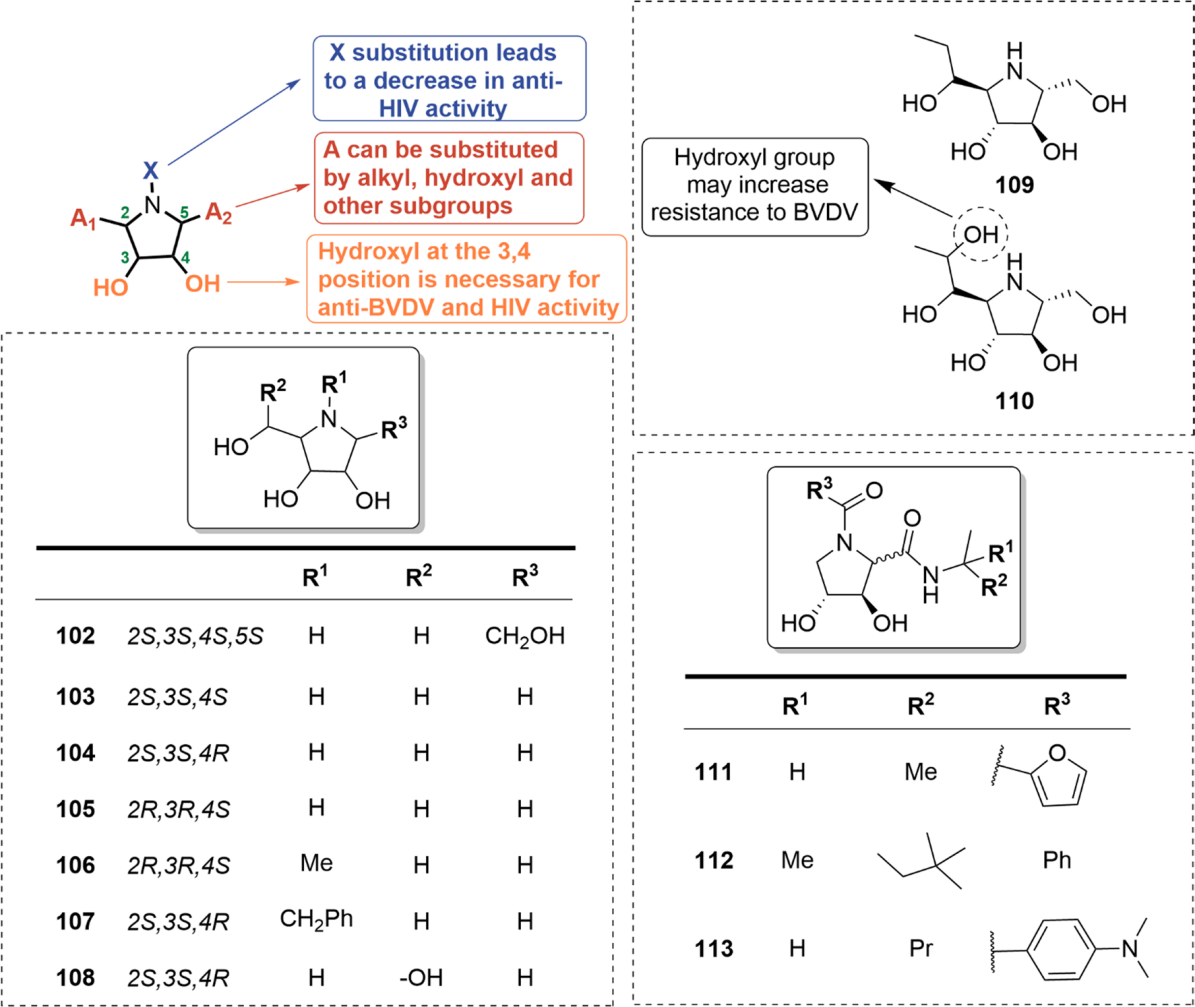
SARs of antiviral pyrrolidine-type iminosugars

Pyrrolidine-type iminosugars, featuring a five-membered ring, mimic the oxocarbenium transition states in a flattened half-chair conformation during glycosidic hydrolysis. These compounds show promising therapeutic potentials against HIV and BVDV infections. Compared to the piperidine-type iminosugars, the structural modifications of pyrrolidine-type iminosugars are more uniform. Current SAR studies primarily focus on manipulating the conformation and substitutions at C2 and C5 positions of the pyrrolidine ring (Fig. 11).

As a typical example, azafructose (i.e., **102**) showed remarkable inhibitory effects against HIV. Removing the hydroxyl group at C1 resulted in a 50% reduction in anti-HIV activity (i.e., **103**) [84]. Stereochemistry also significantly influences the anti-HIV activity, as indicated by diastereoisomers **104** and **105**. Besides, different substituents at N-position have varying impacts on anti-HIV activity. For instance, a methyl substituent at the N position of **105** (i.e., **106**) resulted in a halving of activity, while the benzyl substituent retained the potency (i.e., **107**). Substituents at C4 position did not affect activity (i.e., **108** vs **104**) (Table 6) [53].

**Table 6:** The anti-HIV activity (cytopathic effect, CPE) of **102**–**108** at 0.1 mg/mL in T cells ^84^

Entry	CC_50_ (mg/mL)	CPE (%)
102	> 0.10	25
103	> 0.10	50
104	> 0.10	25
105	> 0.10	50
106	> 0.10	25
107	> 0.10	25
108	> 0.16	25

Additionally, pyrrolidine-type iminosugars have also been found to inhibit BVDV. Modifications at the hydroxyethyl groups at C1 and C4 positions of **102** have shown anti-BVDV activity (i.e., **109** and **110**) [84]. Introducing a carbonyl group at the N position led to promising anti-BVDV activities, where variations in substituents exerted little impact on the antiviral activity (i.e., **111–113**, EC_50_ = 25–30 μM) [85].

Overall, the configuration of substituents on the pyrrolidine ring plays a crucial role in determining the type and effectiveness of antiviral activity. The hydroxyl group at C2 and C3 positions seems to be essential for the anti-HIV and BVDV activities, while substitutions at C1 and C4 positions result in varied outcomes depending on the specific substituent.

### Polycyclic iminosugars

**Fig. 12:**
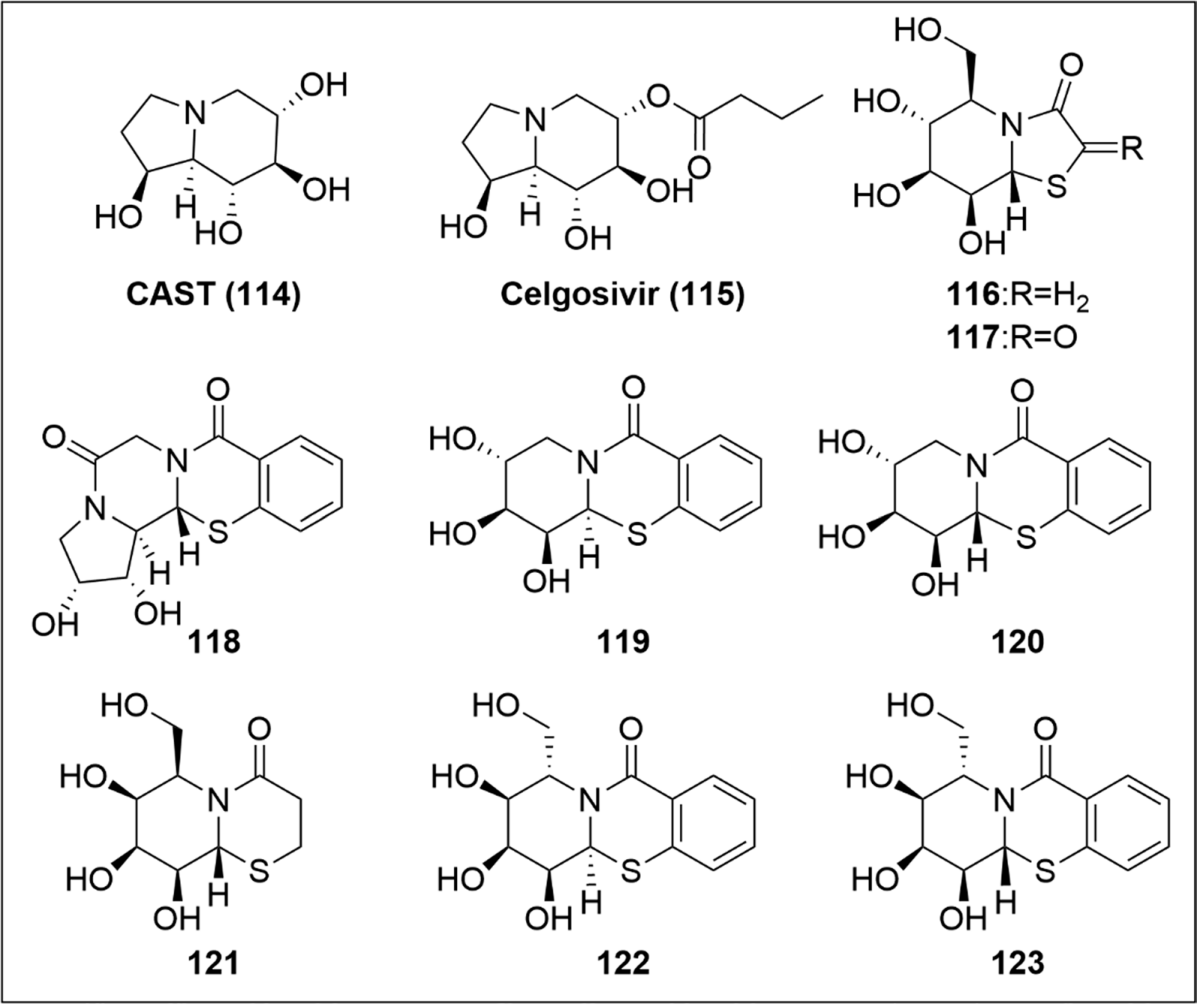
SARs of antiviral polycyclic iminosugars

Polycyclic iminosugars feature an indolizine core with four hydroxy groups at C1, C6, C7 and C8 positions (i.e., **114–123**, Fig. 12). Polycyclic iminosugars provides more opportunities for diverse substituent variations, rendering them promising for further antiviral drug development. A representative compound is castanospermine (CAST, **114**). The hydroxy groups of CAST mimic glucose in pyranose form to inhibit glycosidase. It’s prodrug (i.e., celgosivir, **115**) exhibits improved bioavailability.

There is an overlap in the antiviral spectrum between CAST and celgosivir due to their structural similarities, albeit with varying degrees of activity and selectivity. For instance, both CAST and celgosivir have been shown to decrease SARS-CoV-2 spike protein levels in infected cells and inhibit virus replication. Celgosivir prevented SARS-CoV-2-induced cell death, and reduced viral replication and spike protein levels in a dose-dependent manner in Vero E6 cells [32,35]. Furthermore, celgosivir interfered the N-glycan processing of ACE2, thereby impeding membrane fusion induced by viral envelope proteins. These effects extended to IAV envelope glycoprotein and human coronavirus NL63 (HCoV-NL63) spike glycoprotein lentiviral particles [86]. CAST has shown the capability to decrease neuraminidase (NA) expressions by 50%, thereby impending influenza A virus release and virus production [87].

Notably, CAST and celgosivir exhibit significant variability in activity against certain viruses. This difference in may stem from the increased liposolubility of celgosivir, facilitated by its prodrug strategy, which enhances its bioavailability.

In the context of flaviviruses, CAST exhibited a dose-dependent response against all serotypes of DENV and has undergone Phase I clinical trials for the treatment of DENV infections [88–90]. Compare to CAST, celgosivir demonstrated ten-fold more potent anti-DENV-2 activity (EC_50_ = 0.2 μM), reduced viraemia levels, and enhanced immune responses [91]. Additionally, celgosivir exhibited greater potency against ZIKV by reducing ZIKV RNA and titers in Vero and CHME3 cells without adversely affecting the host cells [92,93]. Besides, celgosivir demonstrated better anti-HIV efficacy compare to CAST, it impairs the processing of viral glycoproteins, resulting in the expression of abnormal gp120. While this did not affect virus production, virus particles were less infectious, which was partly related to reduced CD4 + T cells binding capacity [94].

Furthermore, other more intricate polycyclic iminosugars have emerged as promising candidates targeting HIV reverse transcriptase (HIV-RT) (Table 7). The ring 1 of CAST can be replaced by thiazole rings, as demonstrated by novel bi-/tricyclic thiazolidin-4-one and benzothiazin-4-one-fused iminosugars [95–97]. These compounds maintained the activity against HIV (i.e., **116** and **117** [96,97]), and the effect seemed to be better than the positive control zidovudine (AZT). Ring 1 could also be turned into a six- membered ring (i.e., **121**), which still had inhibitory activity. When the benzene ring was incorporated enhanced anti-HIV-RT activity was observed (i.e., **118** [98], **119, 120, 122, 123** [99]). However, different conformational arrangements of the substituents had a negligible effect (Table 7).

**Table 7:** The anti-HIV-reverse transcriptase activities of **116**–**123**

Entry	IC_50_ (μM)^a^	References
116	0.80 ± 0.30	[99]
117	12.50 ± 1.30	[99]
118	0.82 ± 0.27	[98]
119	1.58 ± 0.29	[99]
120	3.83 ± 0.62	[99]
121	1.41 ± 0.31	[99]
122	0.49 ± 0.09	[99]
123	1.32 ± 0.26	[99]
AZT	18.22 ± 1.59	[99]

^a^The activities were measured with an in vitro HIV-1 reverse transcriptase Kit

Taken together, polycyclic iminosugars exert their antiviral effects through multiple mechanisms, affecting various stages of viral replication, glycosylation, immunity, and protein functions. These versatile compounds hold promise as antiviral agents against diverse enveloped viruses.

## Iminosugars in pre-clinical studies

Iminosugars, characterized by their novel structures and unique mode of actions, hold remarkable promise for the development of innovative anti-virals. The preclinical studies of several iminosugars have provided invaluable insights into their efficacy and safety profiles, as well as pharmacokinetic properties, thereby accelerating their translation from bench to bedside.

### The toxicity and side effects

Due to their host-targeted modes of actions, iminosugars pose significant safety concerns including whether inhibiting α-glucosidases will have harmful effects on the host. In fact, when infected cells are incubated with glucosidase inhibitors, viral yield is significantly reduced, while the general maturation of host proteins in the ER remains unaffected [100]. During acute viral infections, the rapid synthesis of viral glycoproteins makes enveloped viruses more susceptible to ER α-glucosidase inhibition compared to host cells [101]. This differential sensitivity suggests a therapeutic window where partial inhibition of the enzyme can achieve antiviral effects without significantly affecting the host [80]. Certain studies suggest that the strict lattice structure of viral particles may not tolerate minor conformational changes within a single molecule, contributing to this heightened sensitivity [102].

It is noteworthy that most normal proteins appear to tolerate the inhibition of glucosidase activity well, as many proteins can fold efficiently without interaction with calnexin/calreticulin, partially due to the upregulation of other classes of ER chaperones [103,104]. In cases of acute viral infection, adverse effects of glucosidase inhibition, such as ER stress from protein accumulation, are likely transient and reversible upon drug withdrawal. These side effects are relatively mild compared to the impact of the viral infection itself.

Notably, DNJ has demonstrated no significant toxicity in mice at the dosage up to 4000 mg/kg [105]. Besides, it also does not induce general toxicity or genotoxicity in various assays, including bacterial reverse mutation tests, bone marrow micronucleus assays, and spermatid malformation assays, indicating the remarkable tolerability [106].

Beyond glucosidase inhibition, iminosugars may present additional potential adverse effects, necessitating comprehensive investigations into their toxicity and potential side effects. Patients enrolled in the advanced HIV (ACTG 100) reported common side effects such as diarrhea, weight loss, and flatulence when treated with NB-DNJ [107,108]. Oral administration of NB-DNJ also induced reversible sterility and reduced fertility in male mice in the reproductive and developmental toxicity study [109,110]. Similarly, NB-DGJ has shown the reproductive toxicity too, achieving antiviral effects with adequate plasma levels (50 μM), while causing reversible infertility at a dosage of 150 mg/kg daily. NB-DGJ inhibited CD rat testicular β-glucosidase 2 (GBA10) activity at 2 μM without affecting testicular ceramide-specific glucosyltransferase (CGT) at doses up to 1000 μM [111,112].

The safety profile of MON-DNJ was evaluated in randomized, double-blind studies involving single oral doses of up to 1000 mg, demonstrating its safety and tolerability in humans [83]. No cardiovascular, neurological, or respiratory side effects were reported [113]. However, pre-clinical assessments revealed reproductive and developmental toxicity, including visceral and skeletal malformations and increased risk of male infertility. High doses or prolonged exposure to MON-DNJ led to dose-limiting gastrointestinal side effects [113,114]. Similarly, UV-12B treatment in virus-infected guinea pigs resulted in early mortality across all dosage groups (4, 6, 10 mg / kg) [115].

For polycyclic iminosugar, CAST exhibits inherent side effects, including weight loss, lethargy, and dose-dependent thrombocytopenia. The highest intraperitoneal dose of CAST can result in lymphoid depletion in the thymus, spleen, and lymph nodes [116]. Its pro-drug celgosivir exerted enhanced safety. Celgosivir has been proven safe and well-tolerated in dengue patients upon oral administration [89,90], characterized by no inhibition of intestinal disaccharidases, improved oral tolerance, and reduced intestinal discomfort and diarrhea. Nonetheless, diarrhea and flatulence were still observed in HCV-1 patients in a clinical trial [117].

In addition, the five-membered cyclic iminosugar (i.e., **110)** have demonstrated low toxicity at higher doses (200 μg/mL) and specific inhibition of ER glucosidase, thereby sparing the gastrointestinal tract. These compounds hold potential for co-administration with other therapeutic agents to mitigate side effects, such as peripheral neuropathy, hepatotoxicity, osteoporosis [84].

As mentioned above, the most common side effect of iminosugar is gastrointestinal discomforts, including diarrhea. As inhibitors of a broad spectrum of glycosidases and glycosyltransferases [4], iminosugars also inhibit intestinal α-glucosidases (i.e., salivary amylase and pancreatic amylase), interfering with the metabolism of carbohydrates and affecting physiological processes. These effects occur not only in gastrointestinal digestion but also in lysosomal catabolism and post-translational modification of glycoproteins. Inhibition of liver ER and lysosomal glycosidases is sometimes responsible for the development of severe liver dysfunction.

Strategies have developed to address these issues, novel drug delivery systems and stereochemical modifications offer promising solutions. For instance, encapsulation of iminosugars in liposomes significantly reduces the required dosage, thereby enhancing antiviral efficacy while concurrently minimizing toxicity [118]. Furthermore, increasing the size and hydrophobicity of the *N*-alkyl substituent substantially reduces inhibition against intestinal digestive glycosidases (i.e., sucrase, isomaltase). The *N*-alkyl ether chains further increase the potency. Interestingly, altering the stereochemistry from D to L-ido in DNJ derivatives resulted in increased GCS inhibitory activity, while causing a complete loss of α-glucosidase inhibitory activity [119]. This highlights the potential of stereochemical modifications of iminosugars in fine-tuning their glycosidase specificality. Moreover, epimer of AMP-DNM (i.e., L-ido-AMP-DNM) displayed minimal impact on intestinal glycosidase activity but retaining GCS inhibitory activity (IC_50_ = 0.1 µM) [120].

These findings suggest that stereochemical modifications can affect the selectivity of the enzyme inhibitory activity of iminosugars. However, the selectivity of iminosugars has not been thoroughly explored. Further studies are warranted to elucidate the specific inhibition of iminosugars against various glycosidases and glycosyltransferases, thereby paving the way for the development of novel selective antiviral agents.

### Pharmacokinetic studies

Understanding the pharmacokinetic profiles is essential for assessing the antiviral efficacy and safety of iminosugars. Typically, iminosugars exhibit excellent water solubility and chemical stability, along with low metabolic activity. The long fatty acid side chain plays an important role in uptake and retention. For instance, NN-DNJ retains longer in liver than NB-DNJ, which may result from the preferential uptakes of NN-DNJ into hepatic cells [121].

Current pharmacokinetic studies of iminosugars predominantly focus on DNJ [122–124], *N*-Me-DNJ [61], and miglitol. For instance, *N*-Me-DNJ exhibits minimal plasma protein binding and rapid clearance from plasma, primarily excreting through the renal system, with only a small fraction absorbed after oral administration [125]. Miglitol exhibits improved absorption compared to DNJ, but underwent rapid elimination [126,127]. In Fabry patients, NB-DGJ (lucerastat, administered at 1000 mg b.i.d. for 12 weeks) has been found to be well-tolerated, with no observed clinically relevant safety concerns [128]. MON-DNJ has proven to be intact upon incubation with liver microsomes and did not inhibit five most common cytochrome (CYP) isoenzymes, indicating its favorable ADMET profiles [70].

It is essential to recognize the differences in species sensitivity to the *N*-linked glycan processing. These variations significantly impact the anti-EBOV efficacy of iminosugars across different variants. In an anti-filovirus study, pharmacokinetic studies in rhesus monkeys have revealed a tolerance level 7–11 times higher than that observed in rodents [115].These underscore the necessity of understanding pharmacokinetic parameters across different species to optimize the effectiveness of antiviral therapies.

## Conclusion and perspectives

Iminosugars, renowned for their potent biological activities, have served as invaluable tools in exploration nascent glycoprotein processing and maturation. They hold promise as novel therapeutic agents for various clinical settings. Despite a notable surge in iminosugar studies in recent years, their development as anti-virals has yet to reach fruition, with none currently available in clinics. On account of several issues, including gastrointestinal side effects and reproductive toxicity, need to be addressed.

In addition, there is also an urgent call for improving the range of antiviral effects. While host-targeted strategy often lauded for their broad-spectrum antiviral effects, differences in the effects of species of iminosugars on various viruses, and even on different subtypes of the same virus, have been observed. Unfortunately, current understanding in this field remains limited.

In summary, the host-targeting strategy represents a promising yet challenging approach for the development of broad-spectrum anti-virals. Compounded by intricate SARs, the path to practical application requires thorough evaluations of safety and efficacy. These considerations should serve as guiding principles for future studies in the realm of iminosugar-based anti-virals.

## Data Availability

All the data and materials provided in this manuscript are obtained from included references and available upon request.
